# HbAHP-25, a peptide designed *in silico*, exhibits potent anti-HIV activity *in vitro*

**DOI:** 10.1186/1471-2334-14-S3-E30

**Published:** 2014-05-27

**Authors:** Tahir Bashir, CS Kumar, KVR Reddy

**Affiliations:** 1 Molecular Immunology & Microbiology (MIM), National Institute for Research in Reproductive Health, Mumbai, India; 2 Department of Bioinformatics, Dr. D.Y. Patil University CBD Belapur, Navi Mumbai, India

## Background

Identifying and / or designing molecules that can inhibit HIV infection and be safe to the host cells is highly desired.

## Methods

HbAHP-25 was designed *in silico* against CD4 binding domain of gp120 of HIV-1 by molecular docking using Z dock and PROSA softwares. ELISA and SPR were used to determine the binding ability of HbAHP-25 to gp120. Anti-HIV activity of this peptide was checked by two different assays, viz: a) On TZM bl cells, using luciferase assay; b) On CEM-GFP cells and PBMCs using p24 antigen assay. MTT assay, TER/microsphere assay and Immunofluorescence were used to determine the effect of HbAHP-25 on cell viability, epithelial monolayer integrity and permeability.

## Results

Five peptides were designed, and one of the peptides, HbAHP-25 , exhibits significant anti-HIV activity against various strains of HIV-1, such as HIV-1 Ada, HIV-1 NL4-3, and HIV-1 IIIB. ELISA and SPR revealed a direct interaction between HbAHP-25 and gp120, thereby inhibiting its interaction with CD4 receptor. The peptide didn’t affect cell viability even at higher concentrations; nor did it affect epithelial monolayer integrity or permeability. HbAHP-25 also did not interfere with any tight junction proteins such as ZO-1 and Clauddin-1, thus maintaining cell integrity as well.

## Conclusion

The peptide has potent anti-HIV activity, and can be explored as a potential therapeutic /prophylactic/preventive agent.

